# A matched cohort comparison of mTHPC-mediated photodynamic therapy and trans-oral surgery of early stage oral cavity squamous cell cancer

**DOI:** 10.1007/s00405-012-2104-6

**Published:** 2012-07-07

**Authors:** Baris Karakullukcu, Sharon D. Stoker, Anne P. E. Wildeman, Marcel P. Copper, Maarten A. Wildeman, I. Bing Tan

**Affiliations:** 1 Department of Head and Neck Surgery and Oncology, Netherlands Cancer Institute/Antoni van Leeuwenhoek Hospital, Plesmanlaan 121, 1066 CX Amsterdam, The Netherlands; 2 University Medical Center Utrecht, Utrecht, The Netherlands; 3 St. Antonius Hospital, Utrecht, The Netherlands

**Keywords:** Photodynamic therapy, Oral cavity cancer, Transoral surgery

## Abstract

Photodynamic therapy (PDT) of early stage oral cavity tumors have been thoroughly reported. However, statistical comparison of PDT to the surgical treatment is not available in published literature. We have identified and matched cohorts of patients with early stage oral cavity cancers undergoing surgery (*n* = 43) and PDT (*n* = 55) from a single institute experience. The groups are matched demographically and had the same pre-treatment screening and follow-up schedule. Both groups consisted only of tumors thinner than 5 mm to ensure comparability. The endpoints were local disease free survival, disease free survival, overall survival and response to initial treatment. Local disease free survival at 5 years were 67 and 74 % for PDT and surgery groups, respectively [univariate HR = 1.9 (*p* = 0.26), multivariable HR = 2.7 (*p* = 0.13)]. Disease free survival at 5 years are 47 and 53 % for PDT and surgery groups, respectively [univariate HR = 0.8 (*p* = 0.52), multivariable HR = 0.75 (*p* = 0.45)]. Overall survival was 83 and 75 % for PDT and surgery groups, respectively [(univariate HR = 0.5 (*p* = 0.19), multivariable HR = 0.5 (*p* = 0.17)]. In the PDT group, six patients (11 %) and in the surgery group 11 patients (26 %) had to receive additional treatments after the initial. All of the tested parameters did not have statistical significant difference. Although there is probably a selection bias due to the non-randomized design, this study shows that PDT of early stage oral cavity cancer is comparable in terms of disease control and survival to trans-oral resection and can be offered as an alternative to surgical treatment.

## Introduction

Photodynamic therapy (PDT) of early stage oral cavity malignancies has been reported by several authors with varying degrees of clinical effectiveness [1–5]. Recent studies of PDT of carefully selected early stage oral cavity cancers report considerable success with local control of disease [6, 7]. However, there is no published attempt to compare PDT to the golden standard treatment of oral cavity cancers, which is surgery with or without radiation therapy [5]. Although, possible comparing results of surface PDT to published reports of surgery should be done very cautiously [5]. PDT is used to treat a specific subgroup of oral cavity tumors. Only tumors of a certain thickness are treated with PDT due to limitations of light penetration in tissues [7]. There is information in literature comparing surgical treatment of oral cavity tumors with different depths of invasion [8–10]. However, these articles concentrate more on the risk of regional lymph node metastasis rather than prognosis [8–12]. Furthermore, the definition of depth of invasion does not necessarily mean the thickness of the tumor, which is relevant in case of PDT. Therefore, a direct comparison of published data poses a considerable problem.

The ideal manner to compare PDT to surgery is a prospective randomized study. The main problem with such a design is to offer patients a trial where two very different techniques are going to be used, and there is no option to choose which treatment they want. In our institute, PDT is offered routinely to patients with thin oral cavity cancers as an alternative to surgery. By screening our database, we were able to identify comparable cohorts of patients who have had the same tumor work-up including measurement of the thickness of the tumor, management of the neck and follow-up schedule. Comparison of these cohorts gives an idea about the success of PDT compared to surgery of early stage oral cavity tumors.

## Patients and method

In our institute, patients with early stage oral cavity malignancies are evaluated by biopsy of the tumor, ultrasound (US) of the oral tumor, US of the neck coupled with fine needle aspiration biopsies of suspicious nodes, and MRI of the neck. Patients with tumors without detectable neck node metastasis and tumor thickness of less than 5 mm as measured by US are offered to decide between trans-oral resection and mTHPC (*meta*-tetrahydroxyphenylchlorin) mediated PDT. The 5-mm tumor thickness is dictated by the penetration of the light used during mTHPC mediated PDT, which is estimated to be 10 mm, allowing 5 mm of extra safety treatment margin. The neck nodes are monitored with US and FNA as necessary every 3 months for 1 year following the treatment of the primary tumor [13].

We have employed strict selection criteria to identify comparable groups of patients treated between 2000 and 2008 (Table 1). Early stage squamous cell cancers of the oral cavity (Stages I and II) treated with surface PDT and trans-oral surgery were identified. Patients with neck node metastasis detected before the initial treatment, patients undergoing elective neck dissection, resection via a mandibular split or pull-through approach and free flap reconstruction were excluded. The pre-treatment imaging is reviewed to ensure that tumor thickness is less than 5 mm. The pathology specimens of the resected tumors were checked to confirm that tumor thickness were less than 5 mm.

**Table 1:** Inclusion criteria

Inclusion criteria
Squamous cell carcinoma of the oral cavity
T1/T2 primary tumor
Tumor thickness less than 5 mm as determined by ultrasound or tumor not detectable on imaging
No neck as determined by ultrasound and fine needle aspiration as indicated
No elective neck dissection performed
Surgery group: the tumor is removed by trans-oral surgery without mandibular split or pull-through approach
Surgery group: no free microvascular flap reconstruction performed
PDT group: only surface illumination performed (no interstitial illumination)
At least 2 years of follow-up

The primary endpoint was local disease-free survival. Secondary endpoints were disease free interval, response to initial treatment and overall survival. Response to initial treatment is evaluated based on the necessity to perform additional local treatment within the first 2 months after the initial treatment.

### Statistical method

Response to initial treatment was compared using Fisher exact tests. Local recurrence free interval was taken as time until first local recurrence; disease-free survival was taken as time until first local, regional or distant recurrence or death; overall survival was taken as time until death. In all cases, time was taken from final treatment. For local recurrence free interval, patients were censored at time of regional or distant recurrence. Survival curves are presented using the Kaplan–Meier method and compared using univariate and multivariable Cox proportional hazards models. The multivariable models included age, gender, stage (I vs. II), location (tongue vs. floor mouth vs. other) and resection (first primary vs. second/third primary) as covariates. The level of significance was set at 0.05 with no adjustments being made for multiple testing.

## Results

Fifty-five patients treated with PDT and 43 patients treated with trans-oral resection were included in the analysis. The PDT and surgery groups were comparable to each other in terms of age, gender distribution, cancer stage and location (Table 2). Majority of the treated tumors were located in the tongue or the floor of mouth. The average tumor thicknesses were 4 mm for both groups. Six patients, three from each group were lost to follow-up after 2 years. These patients were excluded from disease specific survival analysis.

**Table 2:** Demographics

	PDT (*N* = 55)	Surgery (*N* = 43)	Total (*N* = 98)
Age			
Median (Range)	60 (38–92)	60 (44–88)	60 (38–92)
Sex			
Female	22 (40 %)	17 (40 %)	39 (40 %)
Male	33 (60 %)	26 (60 %)	59 (60 %)
Location			
Tong	20 (36 %)	25 (58 %)	45 (45 %)
Floor of mouth	22 (40 %)	12 (28 %)	34 (35 %)
Lip	1 (2 %)	3 (7 %)	4 (4 %)
Cheek	5 (9 %)	2 (5 %)	7 (7 %)
Retromolar trigon	3 (5 %)	1 (2 %)	4 (4 %)
Alveolar process	2 (4 %)	0 (0 %)	2 (2 %)
Hard palate	2 (4 %)	0 (0 %)	2 (2 %)
Depth (mm)			
Median (Range)	4 (2–5)	4 (1–5)	4 (1–5)
Stage			
Stage 1	45 (82 %)	29 (67 %)	74 (76 %)
Stage 2	10 (18 %)	14 (33 %)	24 (24 %)
Primary vs. second primary			
Primary	47 (85 %)	40 (93 %)	87 (89 %)
Second Primary	6 (11 %)	1 (2 %)	7 (7 %)
Third Primary	2 (4 %)	2 (5 %)	4 (4 %)

Local control was achieved in 49 out of 55 patients after one session of PDT (89 %). In the surgery group, after initial surgery local control was achieved in 32 of 43 patients (74 %). The difference is not significant (*p* = 0.07). Additional treatments to achieve local control included surgical excision in five patients and radiation therapy in one patient in the PDT group. Three of these excisions were done through a mandibular split approach and two by trans-oral approach. In the surgery group, eight patients were treated with additional trans-oral resection, one patient with resection through mandibular-split approach and adjuvant radiotherapy and two patients with radiation therapy alone (Table 3). These patients are included in the survival analysis to be able to detect the impact of initial treatment decision.

**Table 3:** Response to initial treatment

Response to initial treatment	PDT	Surgery	
CR, complete response	49 (89 %)	32 (74 %)	81 (83 %)
PR, partial response	6 (11 %)	11 (26 %)	17 (17 %)
Additional treatment (for PR)			
Trans-oral resection	2 (4 %)	8 (19 %)^a^	10 (10 %)^a^
Mandibular split or pull-through approach	3 (6 %)	1 (1 %)	4 (4 %)
Radiation therapy	1 (2 %)	3 (7 %)^a^	4 (4 %)^a^

^a^One patient received combination of surgery and post-operative radiation therapy

**Fig. 1:**
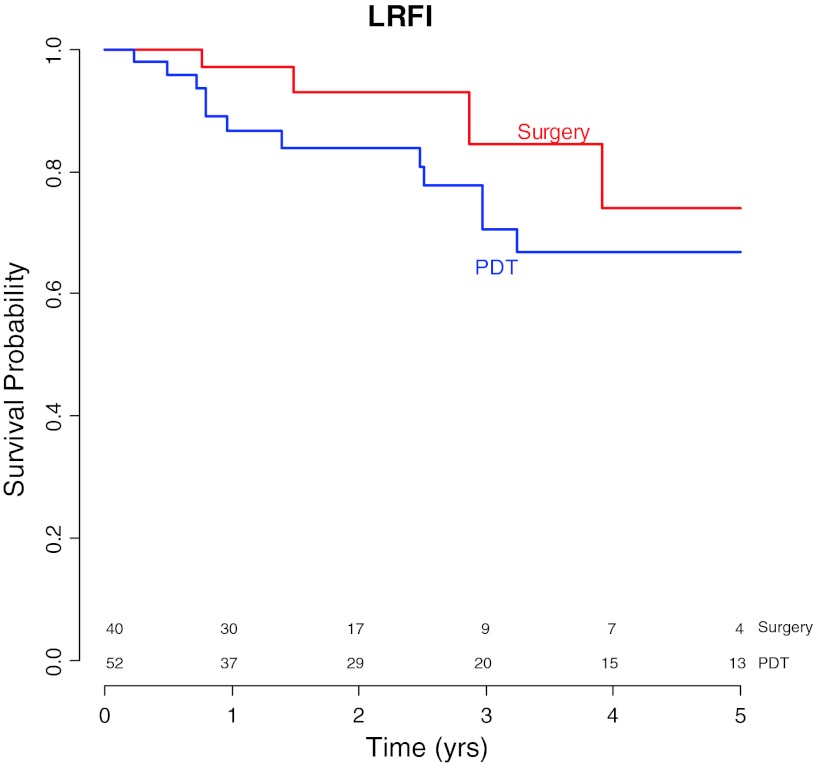
Local recurrence free survival

With the additional treatments included 5-year local disease-free survivals were 67 % (95 % CI 53–85) in the PDT group and 74 % (95 % CI 53–100) in the surgery group. There was no significant difference univariate HR = 1.9 (*p* = 0.26), multivariable HR = 2.7 (*p* = 0.13) (Table 4; Fig. 1).

**Table 4:** Survival analysis overview

	PDT (*N* = 55)	Surgery (*N* = 43)	Total (*N* = 98)
Local disease free survival (LDFI)^a^			
LRFI at 5 years	67 %	74 %	70 %
(95 % CI)	(53–85)	(53–100)	(58–85)
Disease free survival (DFS)^a^			
DFS at 5 years	47 %	53 %	49 %
(95 % CI)	(34–64)	(36–80)	(38–63)
Overall survival (OS)			
Survival at 5 years	83 %	75 %	78 %
(95 % CI)	(72–96)	(61–91)	(69–89)

^a^Six patients were excluded from the LRFI and DFS analyses, three from each group

**Fig. 2:**
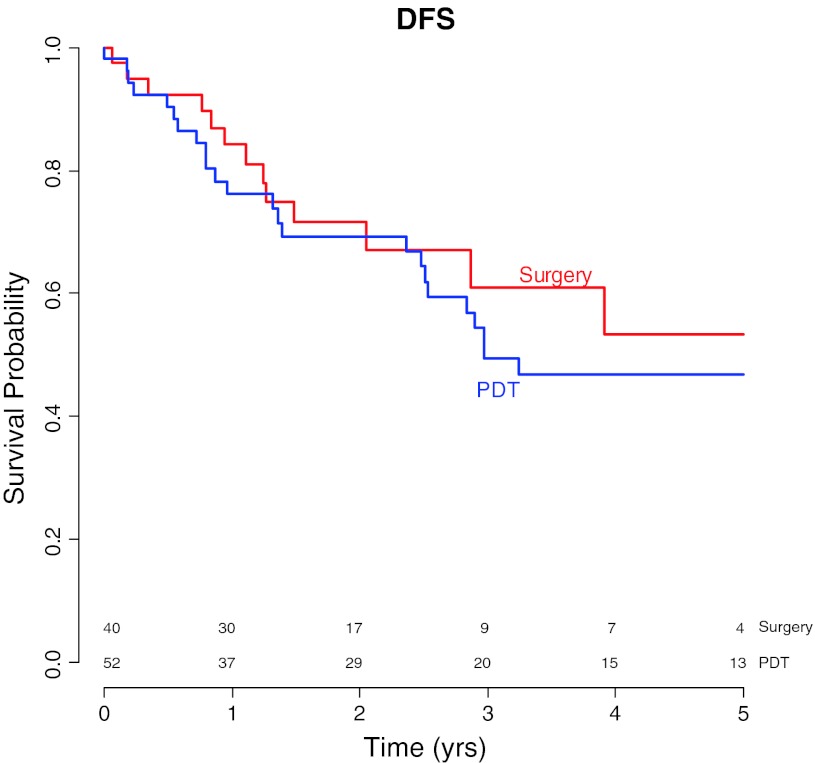
Kaplan–Meier curves for disease free survival

Five-year disease-free survivals were 47 % (95 % CI 34–64 %) in the PDT group and 53 % (95 % CI 36–80 %) in the surgery group. There was no significant difference univariate HR = 0.8 (*p* = 0.52), multivariable HR = 0.75 (*p* = 0.45) (Table 4; Fig. 2).

**Fig. 3:**
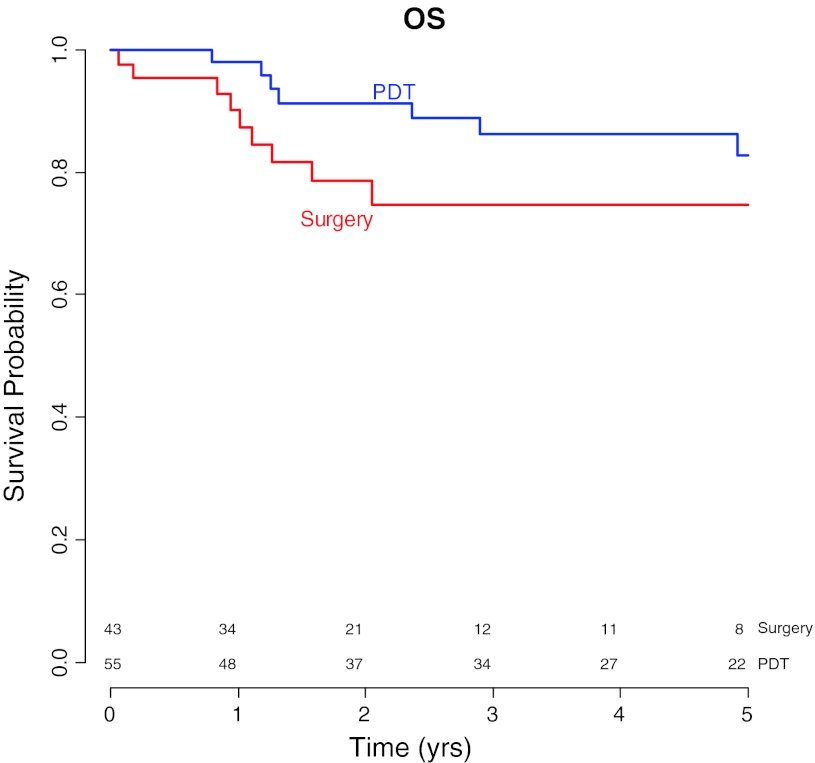
Kaplan–Meier curves for overall survival

The overall 5 year survival was 83 % (95 % CI 72–96 %) in the PDT group and 75 % (95 % CI 61–91 %) in the surgery group. There was no significant difference univariate HR = 0.5 (*p* = 0.19), multivariable HR = 0.5 (*p* = 0.17) (Table 4; Fig. 3).

## Discussion

The results of the current study demonstrate that PDT is comparable to trans-oral resection of early stage oral cavity malignancies in terms of survival and disease control. The non-randomized design of the study introduces a selection bias inherent to this kind of analysis. The selection of the treatment was partly due to patient preferences and partly due to the judgment of the treating physician. While some patients preferred surgery because of reluctance to abide by light restriction measures, others preferred PDT because of its non-invasive nature.

Initial local control was achieved more often with PDT than surgery. This is probably due to larger treatment surface of PDT compared to surgical resections. Surgical resections can be repeated if the resection had insufficient margins or the tumor was not completely removed. Resection and re-resection due to insufficient margins can be considered as one treatment session. However, this approach still has the disadvantage of patient going under general anesthesia twice, whereas PDT could usually be applied under local anesthesia as an outpatient procedure. Being able to detect insufficient local treatment right after surgery and applying re-treatment can be seen as an advantage compared to the wait and watch approach to determine local therapy response of PDT. However, it should be kept in mind that resection with margins is not a guarantee of local control. Hicks et al. [14] reviewed surgery as a single modality treatment of oral cancers. They had no patients with positive margins. Local recurrence was observed in 15 % of patients with close margins (<1 cm) and 9 % of patients with adequate margins (≥1 cm). In our analysis, we have seen no difference in terms of local control between PDT and surgery.

In our study, the computed 5-year disease free survival which includes neck node recurrences is around 50 % for both study groups. This value is in accordance with the disease free survival values reported in the literature (42–72 %) [9–11, 15–18]. This low value of disease control did not reflect to overall survival which remained higher than the literature at around 80 % for both groups. Our approach of routine ultrasonography is probably enabling us to detect lymph node metastasis at an earlier stage [13] facilitating disease control by neck dissection. Whether or not an elective neck dissection should be performed in case of such thin oral cavity tumors is controversial and as of this date no consensus could be reached among the clinicians treating this entity, necessitating a prospective randomized trial [17]. Several articles propose that limited depth of invasion can dictate if elective neck dissection needs to be performed [8–10]. The cut-off value of measured depth of invasion chosen in this analysis can be interpreted as low risk for neck node metastasis.

Although this study has limitations due to lack of randomization and selection bias, it shows that PDT can achieve local control of early stage squamous cell cancer of oral cavity at comparable rates to trans-oral resection. Disease control rates including neck node metastasis are also comparable. PDT is in principle, non-invasive and can be carried out in many instances under local analgesia with lidocain spray. The treatment consists of one session administered at the ambulatory treatment facility. The patients can leave the hospital after an hour of observation. The not-tissue removing approach and ease of application makes PDT more attractive than surgery to some patients. The disadvantage is 2–3 weeks of general light sensitivity which limits patients’ social life.

Photodynamic therapy can be offered as an alternative to surgery to patients with early stage oral cavity cancers, after a careful work-up. Patients who have tumors thinner than 5 mm and no detectable lymph node metastasis are good candidates. Patients who are prone to develop multiple malignancies in the oral cavity such as patients with extensive leukoplakia/erithroplakia are especially good candidates, because of the tissue sparing properties of PDT. The advantages and disadvantages, as well as additional treatments that might be necessary should be extensively discussed with the patient by the physician. The patients should receive light protection measures training by a dedicated nurse. This is essential to prevent any light toxicity at outpatient settings.
